# Efficacy and tolerability of *Helicobacter pylori* eradication regimes in South Kivu, Eastern of the Democratic Republic of Congo: A single center observational study

**DOI:** 10.1002/hsr2.1960

**Published:** 2024-03-07

**Authors:** Tony A. Shindano, Manix I. Masimango, Antoine S. Kishabongo

**Affiliations:** ^1^ Department of Internal Medicine Hôpital Provincial Général de Référence de Bukavu (HPGRB) Bukavu Democratic Republic of the Congo; ^2^ Faculty of Medicine Université Catholique de Bukavu (UCB) Bukavu Democratic Republic of the Congo; ^3^ Faculty of Medicine University of Kindu Kindu Democratic Republic of the Congo; ^4^ Université Officielle de Bukavu (UOB) Bukavu Democratic Republic of the Congo; ^5^ Center for Tropical Diseases and Global Health CTDGH Bukavu Democratic Republic of the Congo; ^6^ Institut Supérieur des Techniques Médicales de Bukavu Sud Kivu Democratic Republic of the Congo

**Keywords:** Democratic Republic of Congo, eradication therapies, *H. pylori*, inflammation

## Abstract

**Background and Aims:**

Treating *Helicobacter pylori* infections has become a major challenge due to increased antibiotic resistance. The aim of this study was to investigate the efficacy and tolerability of the main standard regimens recommended for *H. pylori* eradication in Bukavu, Eastern of the Democratic Republic of Congo.

**Methods:**

The study enrolled patients with evidence of *H. pylori* infection in histological examination or serology testing combined with a positive fecal antigen test. As first‐line treatment, patients were randomized to either a 10‐days (OAC‐10) or a 14‐days (OAC‐14) regimen, employing a combination of omeprazole 20 mg, amoxicillin 1 g, and clarithromycin 500 mg twice daily. In case of failure, a second line regimen was evaluated and included two others protocols: OAC‐10 regimen + levofloxacin 500 mg (OAC‐10+) and the bismuth‐based therapy (pantoprazole + bismuth salt + metronidazole + tetracycline) during 10 days. Our primary endpoint was *H. pylori* eradication and secondarily, the compliance and adverse effects were also evaluated.

**Results:**

A total of 179 patients were enrolled. The eradication rate was 79.2% and 80.5% with the OAC‐10 and OAC‐14 regimen, respectively (*p* = 0.796). Adverse effects were significant higher in the OAC‐14 group than in the OAC‐10 group (36.5% vs. 57.8%; *p* < 0.001). On the other hand, the compliance rate was slightly higher in the OAC‐10 group (97.9% vs. 91.6%, *p* = 0.052) while clinical improvement was almost similar in both groups. Regarding the second line regimen, the bismuth‐based therapy (*n* = 18) seemed to show a better response with 100% of eradication rate and 100% of clinical improvement.

**Conclusion:**

The classic 10‐days triple therapy seems to be as effective as the 14‐days regimen while having in addition a good tolerance. Apart from cost issues, the bismuth‐based therapy seems to be a very good alternative in case of first‐line treatment failure.

## 1 INTRODUCTION

Common infections worldwide include the *Helicobacter pylori*. About 50% of the population of the world is estimated to be infected with up to 80% in developing countries.^1^ *H. pylori* infection is connected to the risk of gastritis, peptic ulcer diseases, and gastric cancer.^2^, ^3^, ^4^

*H. pylori* is a bacillus of helical form that is gram‐negative and thrives in a microaerophilic environment and the bacterium of which resides between the mucus layer and surface epithelial cells in the stomach or any location where gastric‐type epithelium is found. It is detected through gastric mucosal biopsies in patients experiencing upper endoscopy or by non‐endoscopic test, which either identify active infection or detect antibodies. Disease outcome depends on the patterns of *H. pylori* colonization and anti‐inflammation response within the stomach. Short‐lived infection goes with transient hypochlorhydria, enabling the organism to survive in the acidic gastric conditions.

The goal of therapy in an *H. pylori*‐positive patient is to make him symptomless, cure the ulcer, extirpate the infection, and recover the disease. It is widely established that *H. pylori* eradication treatment is associated with reduction of risk of peptic ulcer disease and gastric cancer. Treatment should be well‐endured, efficacious, efficient, and easy to comply with. Drug regimens are expected to have an 80% or 90% eradication rate and should minimize a potential antimicrobial resistance.

A recent consensus stipulates the eradication of *H. pylori* infection combines a proton‐pump inhibitor (PPI) and two or three antimicrobial agents.^5^, ^6^ Although it has evolved over the years, the use of triple therapy containing a PPI, clarithromycin, and amoxicillin was suggested by initial guidelines as the first‐line therapy for 7–14 day duration.^5^

In spite of these recommendations, declining eradication rates relating to antibiotic resistance (especially with clarithromycin) and the potential nonadherence, yield concerns about the efficacy of these regimens. These declining eradication rates are believed to.^7^, ^8^ Hence, for each country, it is recommended to adopt policies based on local antimicrobial resistance profile and efficacy.

Although the DR Congo is one of the countries with high prevalence of *H. pylori* infection, the overall prevalence is not well known. Nevertheless, there are some scarce studies including a community‐based study which had found in 2020 a prevalence of 89% in the city of Bukavu, Eastern of DRC.^9^ Three decades earlier, an endoscopic study has reported a prevalence of 90% in a rural area of the same region.^10^

To our best knowledge, data on bacteriological sensitivity to currently used antibiotic drugs are very scarce. Consequently, there are no national guidelines for *H. pylori* optimal therapy and eradication regimen in the DRC. On the other hand, almost no studies have evaluated the therapeutic efficacy of the main recommended regimens.

Our study aimed at evaluating for the first time the therapeutic efficacy and the tolerability of the main regimens recommended to eradicate *H. pylori* infection in the city of Bukavu, Eastern of DRC.

## 2 MATERIALS AND METHODS

### 2.1 Study design and setting

This anticipated study is conducted in outpatients at the Hopital Provincial General de Reference de Bukavu (HPGRB), from 2018 to December 2022.

### 2.2 Study population and sampling

The study enrolled patients attending the Gastroenterology Unit of HPGRB with upper gastrointestinal symptoms and evidence of *H. pylori* infection. We included all patients aged over 18 years with confirmed diagnosis of *H. pylori* infection during the period of the study.

The exclusion criteria were as follows: patients with allergy to used antibiotics, treated with antibiotics during 4 weeks preceding eradication therapy, pregnant or breastfeeding women, or those without post‐eradication control test.

Our primary endpoint was *H. pylori* eradication and secondarily, the compliance and adverse effects. Adverse effects were self‐reported by patients and ingesting more than 90% of the regimen full dose is good compliance.

### 2.3 *H. pylori* infection diagnosis and eradication confirmation

Diagnosis of *H. pylori* infection was established either by the detection of *H. pylori* on histological examination after endoscopy, either by serology testing when the endoscopy was not performed. In all cases, a fecal antigen test (FAT) was required prior the eradication therapy (TK Biotech). Thereafter, to ensure the eradication of the *H. pylori* infection, we have repeated FAT 4–6 weeks following the completion of the eradication treatment. Hence, successful eradication was confirmed if this repeat test was negative.

### 2.4 Treatments regimens and evaluation of efficacy

Patients were initially selected by simple randomization following the order of arrivals in three different groups of treatment.

Group 1 (OAC‐10): amoxicillin 1 g twice daily + omeprazole 20 mg twice daily + clarithromycin 500 mg twice daily for 10 days.

Group 2 (OAC‐14): amoxicillin 1 g twice daily + omeprazole 20 mg twice daily + clarithromycin 500 mg twice daily during 14 days.

Group 3 (OAM‐10): omeprazole 20 mg twice daily + amoxicillin 1 g twice daily + metronidazole 500 mg twice a day for 10 days.

However, the OAM‐10 protocol was stopped early due to the poor results (<10% of eradication) obtained with the first 15 patients. Hence, the OAC‐10 and OAC‐14 protocols were definitively considered as first‐line therapy regimens and the patients were continuously selected by simple randomization.

Furthermore, for the second line, two others protocols were evaluated, OAC‐10 regimen+ levofloxacin (OAC‐10+) and the bismuth‐based therapy during 10 days. The OAC‐10+ protocol consisted to the addition of levofloxacin 500 mg once daily while bismuth‐based therapy included pantoprazole 20 mg twice daily + bismuth salt 140 mg four times daily + metronidazole 125 mg four times daily + tetracycline 125 mg four times daily for 10 days. To improve the compliance to treatment, molecules of each protocol were provided as combined formulas.

### 2.5 Statistical analysis

For statistical analysis I used the SPSS software version 20.0 (SPSS). The data are presented through standard deviation, frequencies, and percentages. The comparison of categorical data was done through the *χ* ^2^ and Fisher's exact tests. Patients with a compliance less than 80% and those with unknown posttreatment status were excluded from analysis. A *p* value of ≤0.05 was deemed significant.

### 2.6 Ethical considerations

The study was authorized by the ethics committee of the Catholic University of Bukavu. Anonymity was guaranteed during data processing. All patients gave written informed consent about the objectives of the study. It should be pointed out that in the context of the DRC, medical care are largely self‐funded. So medicines were provided to patients from their own pockets.

## 3 RESULTS

### 3.1 Baseline characteristics

**Figure 1:**
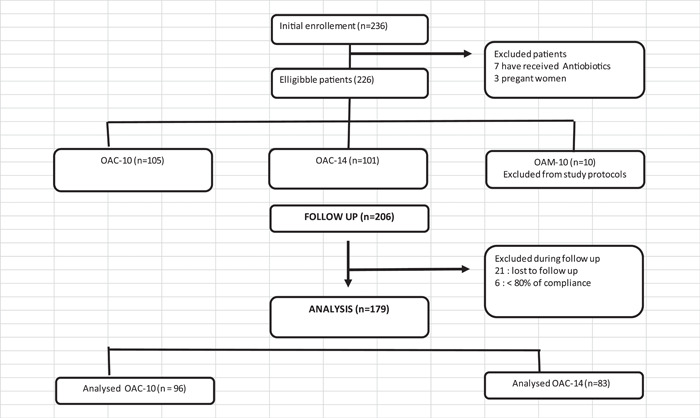
Flowchart for patients inclusion in the first line protocol of treatment.

A total of 179 patients were eligible related to our inclusion criteria. Twenty‐one patients were lost to the follow‐up and six were excluded from the study due to insufficient compliance (Figure 1).

The two treatment groups showed no significant differences in baseline characteristics including age, sex, and initial clinical presentation (Table 1).

**Table 1:** Baseline characteristics of patients eligible for the first line protocol of treatment.

	Total	OAC‐10	OAC‐14	*p*
Number of subjects	179	96 (53.6%)	83 (46.4%)	
Age (mean ± SD)	42 ± 16	41 ± 15	43 ± 16	0.389
Female *n* (%)	124 (64.3)	64 (66.6)	60 (72.3)	0.882
Clinical presentation				
Epigastric pain	167 (93.2)	89 (92.7)	78 (93.9)	0.675
Hematemesis/melena	8 (4.5)	4 (4.2)	4 (4.8)	
Others	4 (2.2)	3 (3.1)	1 (1.2)	

### 3.2 *H. pylori* eradication rates

The eradication rate for the first line protocol of treatment was 79.2% (76/96) and 80.7% (67/83) in OAC‐10 and OAC‐14 group, respectively without significant difference (*p* = 0.796). The rate of clinical improvement was slightly higher in OAC‐10 group (96.9%) than in OAC‐14 (90.4%) but, statistically, this difference was insignificant (*p* = 0.070).

In relation to the second line, the eradication rate was 70.6% in OAC‐10+ group (12/17) and 100% with the bismuth‐based therapy (18/18). The rate of clinical improvement was 64.7% in OAC‐10+ group and equal to 100% during bismuth‐based therapy (Table 2).

**Table 2:** Efficacy of first and second line *Helicobacter* *pylori* eradication therapy.

First line *H. pylori* eradication			
	OAC‐10 (*n* = 96)	OAC‐14 (*n* = 83)	*p*
Effective eradication	76 (79.2)	67 (80.7)	0.796
Subjective clinical improvement	93 (96.9)	75 (90.4)	0.070

Second line *H. pylori* eradication			
	OAC‐10+ (*n* = 17)	Bismuth‐based therapy (*n* = 18)	
Effective eradication	12 (70.6)	18 (100)	0.019
Subjective clinical improvement	11 (64.7)	18 (100)	0.007

### 3.3 Compliance and adverse effects

The comparison of compliance and side effects was only performed with the first line eradication therapy (Table 3). Overall, it was noted that adverse effects event were more frequently reported with the OAC‐10 protocol than with OAC‐14 (*p* < 0.001). The compliance rate was also higher in OAC‐10 group (97.9%) than in OAC‐14 group (91.6%). The difference was at the limit of the significance threshold (*p* = 0.052).

**Table 3:** Comparison of compliance and side effects in patient under first line *Helicobacter* *pylori* eradication therapy.

Variable	OAC‐10 *n* = 96	OAC‐14 *n* = 83	*p*
Compliance (taken 90% of tablets)	94/96 (97.9%)	76/83 (91.6%)	0.052
Side effects			
Diarrhea	1 (2.9%)	3 (6.3)	
Dizziness	2 (5.7%)	2 (4.2)	
Bad taste	15 (42.9%)	22 (45.8)	
Nausea	11 (31.4%)	14 (29.2)	
Skin rash	1 (2.9%)	1 (2.1)	
Vomiting	1 (2.9%)	2 (4.2)	
Weakness	4 (11.4%)	4 (8.3)	
Total side effects % (*n*)	35 (36.5%)	48 (57.8%)	<0.001

## 4 DISCUSSION

This is one of few existing studies on *H. pylori* in the DRC. Rare available data show that it is a common infection with rates approaching 90%.^9^, ^10^ Unfortunately, there is almost no local bacteriological data as well as national policies according to recommendations.^5^ In these conditions, care providers are obliged to use empirical prescriptions without scientific evidence.

In developing countries, bacteriological studies are very difficult to carry out due to the cost and to technical constraints. Existing data are therefore very limited. To fill this gap of information, we therefore wanted to assess the effectiveness of the most prescribed regimens in the city of Bukavu.

Although the choice of the best therapeutic regimen is still on debate, the widely first line regimen remains the triple therapy using amoxicillin, clarithromycin, and omeprazole.^11^ Our study evaluated it as a first‐line protocol and results indicate a success rate of 79.2% when the treatment is given for 10 days and this increases to 80.7% when the regimen is extended to 14 days. This slight increase is not statistically significant and appear to increase rate of adverse events. These findings suggest that these two regimens seem to equal and the conventional tri‐therapy could be shortened to 10 days without losing its effectiveness.

It is classically recommended that the ideal eradication rate should be at least 90% and the therapy must be tolerable with low rate of side effects.^12^ Although low, the rate found in our study is nevertheless acceptable. In fact, the conventional triple therapy is among the first proposed regimens but its efficacy has decreased continuously over the years in several countries.^11^, ^13^, ^14^, ^15^ Overall, the current success rate is around 60% around the world in relation with the increasing of clarithromycin and Imidazole resistance. Thus, this regime is currently only recommended for countries with less than 15% for clarithromycin and 40% for Imidazole.^5^ Due to lack of local bacteriological studies, this standard therapy could still be recommended for our patients given its availability.

Similar rates were observed in Rwanda, a neighboring country close to the town of Bukavu. Investigators found a global eradication rate of 80% using several regimens.^16^ The best rate was obtained with clarithromycin based combination triple therapy with 87% rate of success. This could indirectly suggest a low rate of clarithromycin resistance in the region.

To support a 10‐day regimen, the good compliance and low rate of adverse effects must be emphasized. In low resource country without population health insurance, as the DRC, the best regimen must be both the cheapest and the most effective. On the other hand, the extended treatment exposes to increase number of adverse effects. This could lead to persistence of some digestive complaints.

Nevertheless, the small size of the study population may in part limit the scope of our findings. Thus, our results could not be able to generate any recommendation as they stand. In addition, the single‐center characteristic of our study could also limit the generalizability of the results to the whole country.

Two regimens were compared in the management of patients with eradication failure using the first line therapy. Although small sample sizes, the eradication rate exceeds 90% and reaches 100% with bismuth‐based therapy (*n* = 18).

Kabakambira et al. noted an effectiveness of ciprofloxacin based triple therapy (omeprazole + amoxicillin + ciprofloxacin) close to empiric regimen.^16^ However, it should be noted that their results were reported in patients without prior exposure to any eradication treatment. Their results decrease when the regimen is used as a second‐line regimen. This could suggest the possibility of some degree of fluoroquinolone resistance as previously suspected.^17^, ^18^, ^19^, ^20^, ^21^

Although the recent Maastricht VI/Florence Consensus Report suggests to extend therapy to 14 days to improve eradication rate, it also encouraged to evaluate the effectiveness of 10‐days therapies.^5^, ^18^ That is why this duration was chosen in our study in addition to economic reasons.

In light of our findings, it will be important to evaluate another cost‐effective and alternative treatment to bismuth‐based therapy. Although tolerable and very effective, this latter treatment is not currently available in Bukavu. During our study, patients ordered it from their own pockets in Kinshasa (2000 km of Bukavu). In addition to its unavailability, this combination is also very expensive in relation to the local average income.

Recent studies have assessed potential alternatives in second‐line eradication treatment and the majority seem to point toward the bismuth therapy superiority.^5^, ^22^, ^23^ However, other molecules and therapeutic regimen have shown to achieve relatively satisfactory cure rates in second‐line indication. This includes quinolone‐based triple therapy, sequential therapy, or new antibiotics like Nitazoxanide.^22^, ^24^, ^25^ The most effective rescue regimen remains levofloxacin‐based triple therapy although it did not show satisfactory results in our study. It will therefore be important to continue its assessment with a larger sample, longer duration (14 days) or the use/combination of/with other antibiotics.

Data on bismuth‐based therapy are very rare in Sub‐Saharan Africa but this treatment is no longer available in most developing countries. The search for other therapeutic alternatives is therefore crucial for adapting local guidelines.

### 4.1 Strengths and limitations of our study

To our knowledge, this is a pioneering study in assessing *H. pylori* eradication in the Eastern part of the DRC. Our findings are important because they give an overview of the effectiveness of most used therapeutic regimens. For clinicians, this information is crucial in the absence of local bacteriological sensitivity or resistance data.

The first limitation of our study is the choice of FAT as the outcome measurement test. This poorly sensitive diagnostic was chosen because of limited financial resources and local unavailability of other reference tests (urea breath test or bacterial culture).

It should be remembered that care expenses were fully covered by patients themselves. This situation has limited the randomization of patients during the second line regimen. Indeed, only wealthy patients were able to obtain the bismuth‐based cure.

The second limitation is related to the small size of our sample.

Finally, the clinical response or tolerability of different therapeutic regimens should be considered in taking into account the degree of inflammation of the gastric mucosa. However, this condition was not evaluated in all patients because the endoscopic exploration was not systematic. This undoubtedly explains the effect of PPIs on the improvement of some gastric clinical complaints.^26^, ^27^ This condition should be taken into account when interpreting our data.

All these limitations are to consider in the interpretation of our findings.

## 5 CONCLUSION

The present study results seem to show the efficacy and safety of 10‐days triple therapy regimen using combination of omeprazole amoxicillin and clarithromycin with an average eradication rate of 79.2%. Extending the duration of this treatment to 14‐days showed no significant advantage. Apart from cost issues, the bismuth‐based therapy seems to be a very good alternative in case of first‐line treatment failure.

## AUTHOR CONTRIBUTIONS

**Tony A. Shindano**: Conceptualization; investigation; methodology; writing—original draft; writing—review and editing. **Manix I. Masimango**: Writing—review and editing. **Antoine S. Kishabongo**: Writing—review and editing. All authors have read and approved the final version of the manuscript submitted by the corresponding author (Tony A. Shindano).

## CONFLICT OF INTEREST STATEMENT

The authors declare no conflict of interest.

## TRANSPARENCY STATEMENT

The lead author Tony A. Shindano affirms that this manuscript is an honest, accurate, and transparent account of the study being reported; that no important aspects of the study have been omitted; and that any discrepancies from the study as planned (and, if relevant, registered) have been explained.

## Data Availability

The corresponding author (Tony A. Shindano) had full access to all of the data in this study and takes complete responsibility for the integrity of the data and the accuracy of the data analysis. The data that support the findings of this study are openly available in Mendeley Data: https://data.mendeley.com/datasets/826mv969wn/1
